# The Intricate Interplay between Epigenetic Events, Alternative Splicing and Noncoding RNA Deregulation in Colorectal Cancer

**DOI:** 10.3390/cells8080929

**Published:** 2019-08-19

**Authors:** Raheleh Amirkhah, Hojjat Naderi-Meshkin, Jaynish S. Shah, Philip D. Dunne, Ulf Schmitz

**Affiliations:** 1 Centre for Cancer Research and Cell Biology, Queen’s University Belfast, Belfast BT9 7AE, UK; 2 Nastaran Center for Cancer Prevention (NCCP), Mashhad 9185765476, Iran; 3 Stem Cells and Regenerative Medicine Research Group, Academic Center for Education, Culture Research (ACECR), Khorasan Razavi Branch, Mashhad 9177949367, Iran; 4 Gene & Stem Cell Therapy Program Centenary Institute, The University of Sydney, Camperdown, NSW 2050, Australia; 5 Sydney Medical School, The University of Sydney, Camperdown, NSW 2050, Australia; 6 Computational BioMedicine Laboratory Centenary Institute, The University of Sydney, Camperdown, NSW 2050, Australia

**Keywords:** chromatin remodelling, histone modifications, aberrant DNA methylation, long non-coding RNA, microRNA

## Abstract

Colorectal cancer (CRC) results from a transformation of colonic epithelial cells into adenocarcinoma cells due to genetic and epigenetic instabilities, alongside remodelling of the surrounding stromal tumour microenvironment. Epithelial-specific epigenetic variations escorting this process include chromatin remodelling, histone modifications and aberrant DNA methylation, which influence gene expression, alternative splicing and function of non-coding RNA. In this review, we first highlight epigenetic modulators, modifiers and mediators in CRC, then we elaborate on causes and consequences of epigenetic alterations in CRC pathogenesis alongside an appraisal of the complex feedback mechanisms realized through alternative splicing and non-coding RNA regulation. An emphasis in our review is put on how this intricate network of epigenetic and post-transcriptional gene regulation evolves during the initiation, progression and metastasis formation in CRC.

## 1. Introduction

Besides genomic instability and mutations, the disruption of epigenomic control is a known characteristic of cancer cells [1,2]. It was shown that the development of CRC is driven by an accumulation of genetic and epigenetic aberrations [3].

CRC pathogenesis is associated with three major pathways, which are the basis for molecular subtyping of CRC: microsatellite instability (MSI), chromosomal instability (CIN), and the epigenomic CpG island methylator phenotype (CIMP) [4]. Additionally, an international Colorectal Cancer Subtyping Consortium introduced four consensus molecular subtypes (termed CMS1-4), which are largely based on transcription signalling associated with components of the tumour microenvironment including CMS1 (MSI immune), CMS2 (canonical), CMS3 (metabolic) and CMS4 (mesenchymal) [5]. Although this taxonomy provided a basis for research in the CRC field, increasing evidence has revealed that epigenetic alterations, including DNA methylation, histone marks, chromatin remodelling, and noncoding RNAs (ncRNAs) play pivotal roles in the development of CRC, supporting the idea that epigenetic signatures can be used to further stratify the spectrum of heterogenous CRC phenotypes into molecular subtypes associated with patient prognosis and treatment response.

In this review, we focus on epigenetics, alternative splicing, ncRNAs, and the interplay between these mechanisms of gene regulation in CRC initiation, progression and metastasis.

## 2. Epigenetic Modulators, Modifiers and Mediators in CRC

Epigenetic alterations are strongly associated with neoplastic transformation in CRC [6,7]. Epigenetically induced activation of proto-oncogenes and silencing of tumour-suppressors play a central role in the complexity of cancer emergence, progression and response to treatment [7,8]. Therefore, understanding epigenetic changes as a driving force in colorectal neoplasia opens up new opportunities for the identification of reliable epi-biomarkers and the development of targeted epigenetic therapies in CRCs.

**Figure 1:**
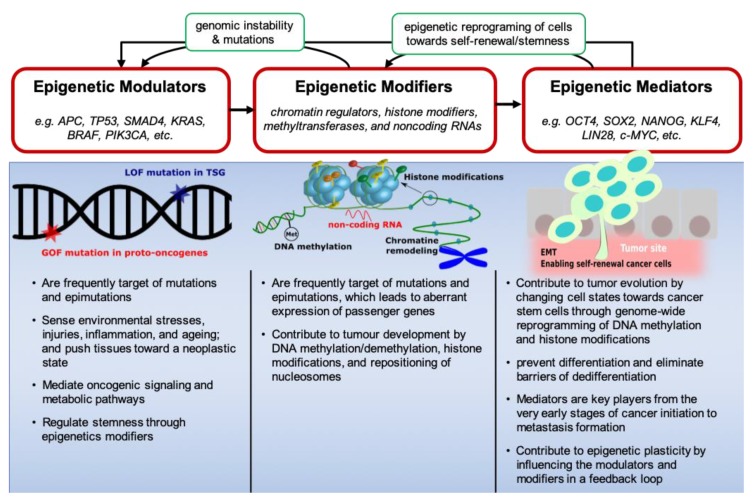
Epigenetic functional system in the initiation and progression of CRC. Environmental cues, such as repeated exposure to carcinogens, inflammation, injury, and ageing impinge on epigenetic modulators. These, in turn, destabilize the epigenome through signalling and metabolic pathways. As a result, chromatin states at epigenetic mediator genes are changed triggering their unscheduled expression. Epigenetic mediators can also influence the plasticity of tumour cells during neoplasia, giving rise to the formation of CSCs and metastases. In all these processes, epigenetic modifiers play a central role. Mutations are frequently seen in epigenome modifying genes and, conversely, the epigenetic changes can cause further mutations and genomic instability in modulators. LOF = loss of function; TSG = tumour suppressor gene; GOF = gene of interest; Met = methylation; EMT = epithelial to mesenchymal transition.

Recently, Feinberg et al. introduced a cancer epigenetics framework in which genes act as epigenetic modulators, modifiers or mediators [9]. In their model, epigenetic modulators regulate the activity of modifiers that, in turn, induce the expression of epigenetic mediators (Figure 1). Mediators dynamically change through feedback loops that target epigenetic modifiers, thereby shaping the Waddington landscape of cancer development in which mediators bring about a cellular transition towards a cancer stem cell state favouring tumour progression [9]. A deregulated epigenome often caused by environmental factors and facilitated by the interplay between modulators, modifiers and mediators can result in the intra-tumoural cellular heterogeneity that enables tumour evolution [9,10].

The same cascade of epigenetic modulators, modifiers and mediators can be documented in CRC. Epigenetic subtypes of CRC are strongly associated with specific somatic mutations that drive the step-wise progression of CRC [6,11,12,13,14]. During the early stages of CRC development, the normal colorectal epithelium is transformed into a benign adenoma by inactivation mutations in tumour suppressor genes (e.g., *APC*, *TP53*, *SMAD4*) and activating mutations in proto-oncogenes (*KRAS*, *BRAF*, *PIK3CA*) [1,15,16]. Subsequently, through the sequential alterations in epigenetic modifiers [17,18] and mediators [19], adenoma progresses to carcinoma and subsequently advances to an invasive and metastatic tumour [6,20]. Therefore, epigenetic modulators, modifiers and mediators work together in generating phenotypic variations of cancerous cells (Figure 1).

Using CRISPR gene editing, Matano et al. created CRC organoids with loss of function mutations in *APC*, *SMAD4* and *TP53*, and gain-of-function mutations in *KRAS* and/or *PIK3CA* that grew independent from niche factors in vitro, and formed tumours after implantation under the kidney sub-capsule in mice [21]. They suggested that driver pathway mutations in epigenetic modulators facilitate the preservation of stem cells in the tumour microenvironment. However, further molecular lesions would be necessary for invasive behaviour. Another study sequentially introduced both inactivation mutations in tumour suppressor genes (*APC*, *TP53*, and *SMAD4*) and an oncogenic mutation in the *KRAS* oncogene to create CRC organoids from intestinal stem cells [21]. By studying this CRISPR-mutated organoid containing four of the most frequently mutated CRC genes, they have demonstrated that quadruple mutants grow independent from niche factors as invasive carcinomas and combined loss of APC and P53 is sufficient for acquiring CIN [22]. Another group has genetically dissected CRC progression (adenoma-carcinoma sequence) by orthotopic transplantation of CRISPR-engineered CRC organoids to study the contribution of common CRC key mutations (in Wnt, EGFR, P53, and TGF-β signalling pathways) to metastasis [23]. Lannagan et al. generated complex preclinical models of serrated CRC by serial introduction of inactivation mutations in five genes (*MLH1*, *TGFBR2*, *RNF43*, *ZNRF3*, and *p16Ink4a*) in BRAF^V600E^ organoids [24]. Although these studies have validated the critical role of mutations in epigenetic modulators in CRC development, emerging studies have shown that mutations in epigenetic modulators such as BRAF^V600E^ and KRAS^G13D^ are tightly connected with the CpG island methylator phenotype (CIMP), which is generated by epigenetic modifiers [13,14,25,26]. These studies support the epigenetic functional framework depicted in Figure 1.

Although the role of *BRAF* and *KRAS* mutations in the development of CRC is well documented, the chicken-or-egg problem for CRC is to definitively prove whether mutations in epigenetic modulators eventually lead to CIMP or CIMP appears first and creates an environment that facilitates mutations in epigenetic modulators. Interestingly, evidence for both hypotheses has been found, suggesting that there are ultimately different pathways for epithelial cells to progress towards cancerous phenotypes in cancer development. The BRAF^V600E^ mutation has been shown to result in CIMP development via increased BRAF/MEK/ERK signalling, which causes MAFG upregulation and phosphorylation. The transcriptional repressor MAFG, in turn, recruits a corepressor complex that includes the chromatin remodelling factor CHD8 and the DNA methyltransferase DNMT3B to CpG islands in the promoters of CIMP genes [25]. In another study, acquisition of the KRAS^G13D^ mutation resulted in the upregulation of zinc-finger DNA-binding protein ZNF304. As a consequence, ZNF304 recruits DNA methyltransferase DNMT1 to CIMP gene promoters causing aberrant hypermethylation [27]. Conversely, other studies have shown that aberrant DNA hypermethylation and CIMP provides a permissive context for mutations in the *BRAF* gene [13,14,26].

Epithelial to mesenchymal transition (EMT)-associated reprogramming of normal and tumour epithelial cells is a result of fundamental changes in several regulatory networks and the interplay between them [28]. Impaired epithelial balance can contribute to the acquisition of a cancerous state, e.g., through the deregulation of epigenetic control mechanisms, the transcriptional machinery, alternative splicing, the expression of non-coding RNAs or alterations in translation and protein stability [28]. Widschwendter et al. showed that cancers may have a stem cell origin in which reversible gene repression normally imposed by an epigenetic modifier (e.g., Polycomb group proteins) is replaced by constant silencing, locking cells into a permanent state of self-renewal that predisposes them to malignant transformation [11]. Another study demonstrated that driver mutations are significantly associated with aberrant DNA methylation in many cancer types, including CRC, and that these epigenetic changes contribute to carcinogenesis. These driver mutation–methylation patterns can be used to classify heterogeneous cancers into subtypes [12]. Recently, an integrative genome-wide DNA methylation and transcriptomic analysis of 216 CRC samples revealed five clinically and molecularly distinct subtypes of colorectal adenocarcinomas, along with an association between genomic methylation and age [26].

Besides genetic instability and mutations, epigenomic disruption can contribute to transformation and the development of cancer-associated phenotypes [1,2]. Understanding the network of epigenetic modifiers provides information to interpret the functional significance of epigenetic drivers of tumorigenesis [2,29]. Epigenetic modifications, as an instructive layer, act on the genome and can be cell type-specific [30,31]. Defects in epigenetic effectors (readers, writers and erasers) mediate the development of cancers, including CRC [1,17,30,31]. Thus, we next focus on epigenetic modifiers and their interactions in CRC cell regulation.

## 3. The Interplay between Non-Coding RNAs and Epigenetics in CRC

The interplay between epigenomics and non-coding RNA (ncRNA) expression and function is currently receiving a lot of attention. Elucidation of this intricate regulatory network between ncRNAs and epigenetic factors may offer new insights into the molecular mechanisms involved in the pathogenesis of CRC and promote accurate diagnostic and prognostic biomarkers, as well as facilitate the development of novel personalized therapeutic approaches. Especially, the ubiquitous functions of long ncRNAs (lncRNAs) in CRC and elsewhere have been subject of many recent reviews [32,33,34,35,36]. LncRNAs have been implicated in diverse biological functions, e.g., acting as a scaffold for interactions between various macromolecules, as a signal for the recruitment of the transcription machinery, as a guide for the localization of ribonucleoproteins or as a decoy for microRNAs (miRNAs) or proteins [37,38]. LncRNAs regulate gene expression at the epigenetic level (by regulating chromatin remodelling, DNA methylation, or histone modification), the transcriptional level (by association with transcription factors, enhancers or promoters), and post-transcriptional level (via alternative splicing, transport and translation of pre-mRNA, or by interacting with miRNAs).

LncRNAs are often dysregulated in the pathological processes of CRC, functioning as oncogenes or tumour-suppressors [3,34,35,39]. For example, lncRNA DLEU1 promotes CRC cell proliferation and migration by recruiting SMARCA1, a subunit of the NURF chromatin remodelling complex, to the promoter of KPNA3, a gene whose expression is associated with a lower survival rate and poorer prognosis in CRC patients [40].

### 3.1. Noncoding RNAs and DNA Methylation in CRC

DNA methylation, in combination with other epigenetic events, has been associated with different phenotypes of CRC including CIN, MSI and CIMP [41,42]. For example, a recent study has shown that global hypomethylation is significantly associated with CIN in sporadic CRC [43]. Another study confirmed the strong correlation between global DNA hypomethylation and CIN but also found that the MSI phenotype correlates with regional hypermethylation [44]. Interestingly, they found that CRC cells with CIN have an open chromatin conformation and are enriched in histone acetylation, both in repetitive elements and coding regions. Conversely, MSI phenotypes have a higher incidence of closed chromatin structures alongside low levels of histone acetylation. This supports the notion that patterns of DNA methylation in combination with other epigenetic changes have an impact on phenotype-specific gene expression and CRC pathogenesis [44].

Aberrant DNA methylation also contributes to later stages of CRC, for example by establishing a CIMP phenotype through global genome hypermethylation, which results in silencing of tumour suppressor genes, such as *CDKN2A/p16* [45]. CIMP has a strong association with the occurrence of somatic mutations at driver oncogene loci, such as BRAF^V600E^ and KRAS^G13D^; supporting the existence of links between genetic mutations and DNA methyltransferases [25,27]. The association of driver mutations, including BRAF^V600E^ and KRAS^G13D^ with methylation patterns can be used to stratify the heterogeneous cell population of a tumour into homogeneous subtypes [12].

Epigenomic and transcriptomic profiling of the colon cancer cell line DLD1 showed that a signature of mRNAs, miRNA, lncRNAs, and epigenetic alterations are associated with CRC metastasis. In particular, global hypomethylation of gene-regulatory regions was found during tumour progression, with the lowest degree of methylation present in metastases-isolated cells. Due to promoter demethylation, the expression of H19 was elevated, a lncRNA which is associated with poor survival [46]. Using a CRISPR loss-of-function screen, McCleland et al. identified a member of the bromodomain and extra terminal (BET) protein family (BRD4) as a key epigenetic regulator, which interacts with lncRNA CCAT1 for BET-mediated c-MYC regulation in CIMP+ CRC; suggesting CCAT1 as a predictor of sensitivity to BET inhibitor drug JQ1 [47]. Chromatin readers such as BET are considered *druggable* targets for cancer treatment [48].

LncRNAs can regulate genome-wide DNA methylation in association with the methyltransferase DNMT1, especially DACOR1 (DNMT1-associated Colon Cancer Repressed lncRNA 1), which has a highly tissue-specific expression in the normal colon [49]. The induction of tumour suppressor DACOR1 in colon cancer cells restores DNA methylation of thousands of CpG dinucleotides at hypomethylated sites in colon tumours including intergenic regions, promoters and gene bodies of oncogenic transcription factors such as FOS and JUN [50].

5-hydroxymethylcytosine (5hmC) is the first oxidative intermediate product of the 5-methylcytosine (5mC) demethylation by the ten–eleven translocation (TET) protein family. Apart from its intermediate role in the cytosine demethylation pathway, 5hmC has multifaceted regulatory functions with emerging importance in cancer [51,52,53,54,55]. Hu et al. indicated that abnormal tumour-specific enhancers and/or promoters modified by 5hmC promote dysregulation of CRC-related lncRNAs such as TCONS_l2_00000584 and LINC00189 [53]. Mechanistically, 5hmC, along with histone marks and transcription factors, determinate open chromatin structures to facilitate long-range chromatin interactions at lncRNA loci and thus regulate lncRNA transcription. LncRNA enhancers marked with 5hmC show higher transcriptional activity than enhancers without 5hmC mark, thereby contributing to the pathogenesis of CRC [53].

Another lncRNA named HIF1A-AS2 plays an oncogenic role by acting as a competing endogenous RNA. HIF1A-AS2 is a decoy for miR-129-5p, whereby it indirectly promotes DNA Methyltransferase 3 Alpha (DNMT3A) expression and positively affects EMT and progression of CRC [56]. Interestingly, miRNA expression is regulated by DNA methylation and histone modifications as well during CRC tumorigenesis. Almost 10% of all miRNAs are regulated by DNA methylation in CRC cells [57]. DNA methylation-mediated repression of three miRNAs (miR-181a, miR-135a and miR-302c) promotes CRCs with MSI and 5-FU resistance according to Shi et al. [58]. MiRNA-132 was proven to undergo transcriptional inactivation by DNA hypermethylation and implies a poor prognosis in CRC [59]. Another study has identified that miR-133b is markedly downregulated through promoter hypermethylation in human CRC tissues compared to healthy colon cells [60].

MiRNAs are also involved in DNA methylation regulation. Low miR-203 expression in CRC, for example, indirectly causes *ABCG2* promoter methylation lowering the expression of this important efflux transporter and thereby CRC development. MiR-203 targets DNA Methyltransferase 3 Beta (DNMT3B), which is relieved from post-transcriptional repression in CRC and can, therefore, methylate the *ABCG2* promoter [61].

An overview of the interplay between ncRNAs and DNA methylation in CRC is provided in Table 1.

### 3.2. Noncoding RNAs, Chromatin Remodeling, and Histone Modifications in CRC

Among many other functions, lncRNAs are involved in the *cis*- and *trans*-regulation of gene expression. For example, the non-coding RNA DLEU1 contributes to CRC development and progression by recruiting SMARCA1, an essential subunit of the NURF chromatin remodelling complex, to the promoter of the *KPNA3* gene. High expression of DLEU1 and KPNA3 correlated with poor prognosis in CRC patients [40].

Several studies have shown that three lncRNAs (CCAT1-l, CCAT1 and CCAT2), located upstream of the proto-oncogene *MYC*, are highly expressed in microsatellite-stable CRC and have been implicated in CRC predisposition by different mechanisms of action [68,69,70,71]. Xiang et al. have shown that CCAT1-L promotes CRC-specific chromatin looping through long-range interactions between the MYC promoter and its upstream enhancers. Additionally, CCAT1-L modulates chromatin conformation at these loop regions by interacting with CTCF [70]. It was shown that upregulation of c-MYC facilitated by a large chromatin loop is linked to a cancer risk-associated single-nucleotide polymorphism (SNP, rs6983267) in CRC cells. Later, Ling et al. demonstrated that the same SNP affects CCAT2 expression and the risk allele of SNP rs6983267 produces more lncRNA CCAT2 transcript, which in turn up-regulates MYC, miR-17-5p, and miR-20a through physical interaction with transcription factor TCF7L2. This results in an enhancement of WNT signalling activity. CCAT2 itself is a downstream target of WNT, suggesting the existence of a feedback loop [68]. Recent studies confirmed that CCAT1 and CCAT2 promote chromosomal instability in CRC pathogenesis and metastasis progression. Both lncRNAs are considered valuable prognostic markers for CRC since high expression of CCAT1 and CCAT2 are associated with cancer recurrence and poor overall survival [69,71].

Epigenetic interplay with lncRNAs also affects advanced stages of CRC. Kogo et al., for example, revealed that the lncRNA HOTAIR, in cooperation with the PRC2 complex widely reprograms chromatin organization and thereby promotes liver metastases in stage IV CRC patients [72].

Besides DNA methylation and chromatin remodelling, histone modifications are also recognized as early epigenetic events in cancers including CRC. Histone modifications recognized as the ‘histone code’ affect chromatin structure and gene expression during tumorigenesis. In an integrated analysis of RNA sequencing data of matched primary tumours, synchronous liver metastases and normal colon tissues, as well as H3K4me3 ChIP-seq data, Chen et al. observed that H3K4me3 was enriched at transcription start sites of lncRNAs dysregulated in CRC [73].

Xu et al. demonstrated that lncRNA SNHG1 directly interacts with the EZH2 subunit of Polycomb Repressive Complex 2 (PRC2) and modulates histone methylation of Kruppel like factor 2 (*KLF2*) and *CDKN2B* promoters in CRC [74]. SNHG1 also functions as a miRNA sponge in the cytoplasm and increases Cyclin D2 (CCND2) expression by sequestering miR-154-5p [74]. We collated other examples of lncRNAs that sponge miRNAs in CRC in Supplementary Table S1. Ding et al. revealed that lncRNA CRNDE (Colorectal Neoplasia Differentially Expressed) epigenetically silences DUSP5 and CDKN1A expression by binding to EZH2, a key component of the PRC2 complex. Thereby, CRNDE contributes to advanced pathological stages in CRC [75].

DNA methylation and histone modifications appear to mutually reinforce silencing of tumour-suppressor genes in CRCs. Wang et al., for example, have found that long non-coding RNA 34 (or Lnc34a) recruits DNMT3A via Prohibitin-2 (PHB2) and Histone Deacetylase 1 (HDAC1) to simultaneously methylate and deacetylate the *MIR34A* promoter [76]. Thereby, miR-34a transcription is epigenetically switched off. Interestingly, microscopy experiments showed that Lnc34a is distributed unevenly when colon cancer cells divide so that the production of miR-34a is asymmetrically inhibited in one daughter cell but not the other. Wang et al. confirmed that Lnc34a is upregulated in late-stage CRCs and overexpressed in cancer stem cells, which helps them to proliferate more rapidly [77].

LncRNA themselves are regulated by histone modifications. The transcription factor Ets-1 negatively regulates BRAF-activated lncRNA (BANCR) expression by binding and deacetylating histone H3 within the *BANCR* promoter during CRC progression [78].

An overview of the interplay between lncRNAs and epigenetic events in CRC is provided in Table 2.

## 4. Regulation of Alternative Splicing in CRC

Many recent studies have shown that alternative splicing (AS) is a key feature for transcriptomic variations in CRC [91,92,93,94,95,96]. AS increases the diversity of both non-coding RNAs (regulatory) and coding RNAs (protein isoforms). These transcriptome variations can be a result of either mutations in or aberrant expression of *trans*-acting splicing factors such as hnRNPL [97], SRSF1 (alias ASF/SF2) [98,99,100], and SRSF6 [101] or mutations in *cis*-regulatory sequences [102,103,104].

Several recent studies have demonstrated that RNA binding motif 4 (RBM4) initiates a hierarchical AS cascade in CRC development; implying that splicing is highly regulated in this process [105,106,107]. Lin et al. demonstrated that RBM4 and PTBP1 exhibit opposite effects on modulating the utilization of splicing factor SRSF3 exon 4. SRSF3, in turn, modulates the metastatic signature of CRC cells, by reprogramming the splicing variants of MAP4K4 with distinct effects on JNK phosphorylation and subsequent downstream signalling pathways [107]. Another study highlighted the potential value of targeting this splicing cascade for CRC treatment and demonstrated how cell migration and angiogenesis are increased via RBM4-regulated isoform expression of Nova1 (exon 4 skipping), SRSF6 (intron 2 retention), and VEGF165 upregulation. RBM4-mediated splicing regulation was shown to promote CRC progression [106].

### 4.1. Epigenetic Regulators of Alternative Splicing in CRC

Epigenetic splicing regulation works through different modes including (1) the modulation of the RNA Pol II elongation rate inducing either exon skipping or inclusion, (2) splicing factor recruitment or sequestration and (3) adaptor/scaffolding function. Modes of epigenetic regulation can lead to changes in splicing factor concentration both spatially and temporally.

#### 4.1.1. Modulation of RNA Pol II Elongation Rate

Several studies have shown that histone marks decorate specific exons to regulate RNA Pol II elongation and thus influence co-transcriptional splicing [108,109,110]. For example, Poly (ADP) ribose polymerase (PARP1) marks histones and thereby changes nucleosome deposition at specific exon-intron boundaries, which in turn affects RNA Pol II movement and finally alters AS decisions in a context-specific manner [110]. Riffo-Campos et al. showed that low nucleosome occupancy, due to differential histone marks at exon 4A of *KRAS*, resulted in an accelerated RNA Pol II elongation rate and subsequent lower abundance of isoform 4A in the CRC cell lines HCT116 and SW48 [109]. Yuan et al. demonstrated in a CRC mouse model that mutation of the histone methyltransferase *SETD2* slows down transcription elongation and thereby facilitates the removal of intron 2 of dishevelled segment polarity protein 2 (DVL2) pre-mRNA; thereby augmenting Wnt/β-catenin signalling and tumorigenesis. Conversely, in normal cells SETD2-catalyzed H3K36me3, thus mediating intron 2 retention in the DVL2 transcript, which induces a premature stop codon. As a consequence, the DVL2 mRNA is degraded via nonsense-mediated decay [111]. Nonsense-mediated decay is a common mechanism of AS-coupled auto-regulation of diverse mRNAs triggered by the retention of an intron harbouring a premature stop codon [106,112].

#### 4.1.2. Splicing Factor Recruitment or Sequestration

Chromatin structures and DNA methylation are important for splice site recognition [113,114,115,116]. Epigenetic events can also help to recruit splicing factors to a specific locus [117,118]. Using ChIP-seq and RNA-seq analyses Kfir et al. revealed that SF3B1, an essential component of the U2 snRNP complex, specifically binds nucleosomes positioned at short exons flanked by long intronic sequences, suggesting that differences in chromatin organization between exons and introns pinpoint splicing factors to their pre-mRNA targets and determine splicing decisions [119]. Kim et al. confirmed that splicing factors specifically bind to post-translationally modified histone residues near exons that are targets of AS [118].

Histone modifications, DNA methylation and epigenetic modifiers can team up to affect AS. Maunakea et al. revealed that alternatively spliced exons are enriched in DNA methylation and that DNA methylation in the gene body can enhance exon recognition by recruitment of the methyl-CpG binding protein 2 (MeCP2) [120]. Similarly, using reduced representation bisulfite sequencing (RRBS) data from primary colon tumours [121], Gelfman et al. confirmed that 5mC signals at CpG sites are accompanied with elevated nucleosome occupancy involved in exon recognition [122]. MeCP2, attracted through DNA methylation, is a writer of histone marks. Other chromatin-binding proteins such as heterochromatin protein 1 (HP1) read these histone marks and act as an adaptor for coupling transcription and AS of nascent pre-mRNA [123]. HP1 not only links histone methylation marks to RNA splicing but also contributes to the progression of CRC by affecting cell cycle-related genes, including *CDK6* and *p21* [124,125]. Davie et al. confirmed that some epigenetic marks such as histone H3K4 trimethylation in coordination with epigenetic writers and readers dynamically affect pre-mRNA splicing and vice versa [126].

Several studies have shown that active cellular mechanisms and mutations can dynamically change histone marks and preferential association of splicing factors with exons [126,127,128]. For example, the G13D mutation in KRAS impacts on the epigenetic modification of heterogeneous nuclear ribonucleoproteins (hnRNPs) that is involved in pre-mRNA splicing [129]. Riffo-Campos et al. suggest that *KRAS* mutations affect AS of *EPDR1* and *ZNF518B* in CRC cells leading to differential isoform expression in these genes [130].

### 4.2. Non-Coding RNAs and the Regulation of Alternative Splicing in CRC

MiRNAs regulate gene expression post-transcriptionally by binding to partially complementary sites in the 3′ untranslated region of their target mRNAs and thereby mediating target degradation or translation repression [131]. The oncogenic miRNAs miR-1298 and miR-92a downregulate the expression of SFQ and RBM4 in CRC [132,133]. Reduced expression of RBM4 leads to increased levels of nPTB and specific exon 10 inclusion. nPTB, in turn, increases FGFR2 IIIc (mesenchymal-specific isoform) and pyruvate kinase M2 (PKM2) transcripts, which result in the progression and metabolic signature of CRC cells [133]. Studies have shown that non-coding RNAs, histone modifications and splicing factors work together to realize the isoform switch of FGFR2 protein from the epithelia-type FGFR2 IIIb to the mesenchymal-type FGFR2 IIIc [133,134,135]. Isoform switching from PKM1 to PKM2 governed by PTBP1 and PTBP1-associated miR-1 and miR-133b is critical for the maintenance of the Warburg effect in CRC cells [136].

The involvement of lncRNAs in the regulation of AS is known and has been reviewed elsewhere [137,138,139]. AS is a highly tissue/cell type-specific process critical to generate protein isoforms. LncRNAs have an important role in the establishment and maintenance of cell type-specific splicing outcomes by interacting with several Polycomb-group proteins, histone modifiers and the chromatin-splicing adaptor complex, as well as splicing factors [134,140]. Thereby, lncRNAs can also contribute to CRC development [141,142]. For example, LINC01133 titrates the splicing factor SRSF6 away from its RNA targets and thereby inhibits epithelial-mesenchymal transition in CRC mouse models [141]. However, the exact underlying mechanism by which SRSF6 controls epithelial-mesenchymal transition and metastasis is yet to be defined. MALAT1 regulates AS by influencing the localization of SR splicing factors into nuclear speckle domains [140]. It thereby regulates cell-type-specific AS in a concentration- and phosphorylation-dependent manner [140,142]. Another study found that MALAT1 promotes tumour growth and metastasis in CRC by competitively binding to SFPQ (also known as Polypyrimidine Tract-Binding Protein-associated splicing factor) and releasing oncogene PTBP2 (polypyrimidine tract binding protein 2) from the SFPQ/PTBP2 complex [143]. Interestingly, MALAT1 and PTBP2 are overexpressed in CRC, but SFPQ remains unchanged in CRC tissues compared to adjacent normal tissues [143]. Another lncRNA named GAPLINC (Gastric adenocarcinoma predictive long intergenic noncoding RNA) can also bind to SFPQ, as well as NONO. SFPQ and NONO promote cell invasion, motility and metastasis in CRC, partly by inducing the expression of snail family zinc finger 2 (SNAI2) [144]. However, the exact underlying mechanisms remain to be elucidated.

Some lncRNAs are encoded on the antisense strand of the gene, whose isoform expression they influence via AS [145,146]. For example, UXT-AS1 promotes CRC progression by changing the expressed isoform of UXT from UXT1 to UXT2 via AS. UXT-AS1 is significantly upregulated in CRC and associated with poor prognosis [146].

We have summarized non-coding RNAs that have an impact on AS in CRC in Table 3.

**Figure 2:**
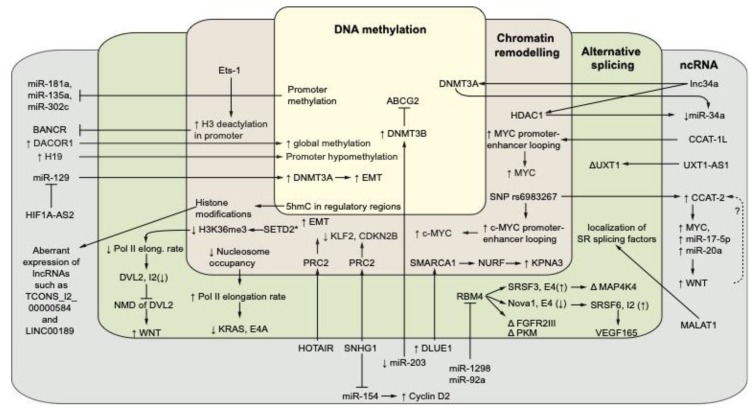
Overview of cross-talk between gene-regulatory layers in CRC. This figure depicts selected examples of the deregulated interplay between epigenetic events, alternative splicing (AS) and noncoding RNA in colorectal cancer. See the text of the manuscript for further details. ↑ and ↓ arrows represent up- or down-regulation or higher or lower activity of a factor, respectively. Intron and exons are abbreviated as E or I, respectively. In the cases of AS, ↑ and ↓ represent increased and decreased usage of an exon or intron, respectively. Δ represents isoform switching of a transcript due to AS. SET2D* represents a mutant of SET2D. The dashed arrow with question mark (?) represents a predicted feedback loop between WNT and CCAT-2. A vector graphics version of this figure is provided as Supplementary Figure S1.

In Figure 2 we summarized current knowledge about the intricate interplay of gene-regulatory mechanisms in CRC across multiple layers.

## 5. Epi-Biomarkers and Promising Targets for the Design of epi-Drugs

Understanding epigenetic regulation of gene and/or protein expression and integration of these factors with genomic data in the context of CRC genesis can help to develop novel epigenetic biomarkers for diagnosis and epigenetic drugs for the treatment of CRC patients. For example, the *SEPT9* gene methylation assay, which aims to detect abnormal methylation at the *SEPT9* promoter region, is the first FDA-approved assay for CRC screening using an epigenetic biomarker [147].

In addition to expression profiling of epigenetic modifiers (e.g., non-coding RNA), the assessment of the epigenetic state (e.g., DNA methylation) of epigenetic modulators and mediator genes can be utilized as prognostic, diagnostic and predictive biomarkers for CRC. Epigenetic modifiers have been shown to act in a cell type-specific manner and some were found highly stable in biofluids. This makes them attractive biomarkers. The biological significance of epigenetic modifiers has already been proven in CRC, and so has their potential to be used as epi-biomarkers for different CRC stages, including the early disease [148,149], EMT [56], metastasis [46], as well as the resistance to drugs [80,150]. Increased levels of lncRNAs such as HOTAIR, DLEU1 and UXT-AS1, which can alter the epigenetic landscape of CRC-promoting genes, have been shown to be associated with poor prognosis in CRC [40,72]. In addition to genome-wide DNA hypermethylation (CIMP phenotype), resulting in reduced expression of tumour suppressor genes, studies have revealed that transcriptional profiling of ncRNAs may offer further molecular stratification and subtyping of CRC patients [151,152]; supporting the idea that epigenetic modifiers should be considered in subtyping strategies.

**Figure 3:**
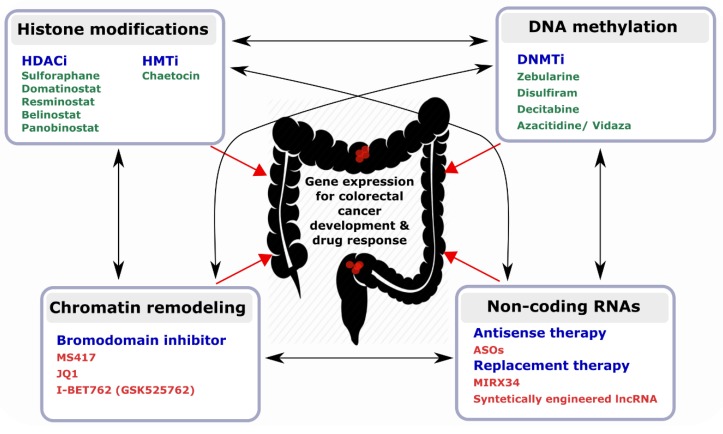
Representative drugs targeting epigenetic modifiers. Green colour represents FDA-approved drugs and red colour represents other potential drugs for targeting epigenetics modifiers. Black arrows indicate the reciprocal interplay of epigenetic events with ncRNAs and red arrows represent their effects on the CRC genome and transcriptome.

Besides their potential as tractable biomarkers in CRC, epigenetic modifiers hold great promise for developing epi-drugs (Figure 3). In contrast to irreversible genetic mutations, epigenetic modifications are reversible and dynamic, thereby making them a promising therapeutic target [153]. Several FDA-approved examples of epi-drugs, such as HDACi and DNMTi, have already been tested in clinical settings [154]. A growing number of studies also support the use of ncRNAs as potential targets for anticancer drugs [155,156]. The first synthetic miRNA mimic to enter a clinical trial was MRX34 (NCT01829971), a miR-34a replacement. The aim of the trial was to evaluate the safety of MRX34 in patients with advanced solid tumours including CRC [157]. Although MIRX34 showed antitumour activity in a subset of patients, the project was terminated because of immune-related serious adverse events reported in five patients treated with MIRX34.

It has been demonstrated that some DNMT/HMT inhibitors and HDAC inhibitors exert their therapeutic effects by modulating the expression of regulatory non-coding RNAs including miRNAs [158] and lncRNAs [53]; implying that the effects between epigenetics modifiers are reciprocal. Even some lncRNAs such as HOTAIR mediate the reciprocal regulation between a histone-lysine N-methyltransferase enzyme (EZH2) and a DNA methyltransferase (DNMT1) [159]. Therefore, certain small molecule compounds such as AC1Q3QWB (AQB) are used in cancer therapy for selectively blocking HOTAIR-EZH2 interaction. The combinatorial use of AQB and 3-Deazaneplanocin A (DZNep; inhibitor of EZH2) offers an even more efficient treatment [160]. Another study has reported the use of an antisense oligonucleotide (ASO) to displace lncRNA XIST, the initiator of X-chromosome inactivation, from inactive X [161]. More ASOs targeting lncRNAs are currently in development [162].

Among the growing number of lncRNAs known to be involved in CRC, it is likely that more links between lncRNAs and epigenetic events will be found (see examples in Table 1 and Table 2), such that combination therapies may provide a more effective treatment option. Such an approach could be achieved using ASOs against a CRC-specific lncRNAs or synthetically-engineered lncRNAs, in combination with current standard-of-care chemotherapies to ensure synergistic effects of epi-drugs.

Of note, many ncRNAs originate from cells in the tumour microenvironment (i.e., fibroblasts, immune cells, endothelial cells) and shuttle to cancer cells via exosomes, providing another option of therapy which has been extensively reviewed in our recently published article [163].

## 6. Future Directions

Colorectal cancer arises in a stepwise mode from either discrete genetic alterations or epigenetic perturbations. Multiple epigenetic mechanisms, including DNA methylation, histone modifications, chromatin remodelling and non-coding RNAs are all involved in CRC pathogenesis. Among these, lncRNAs are emerging as highly versatile players in diverse biological processes regulating gene expression at the epigenetic-, transcriptional, as well as post-transcriptional levels. Although ncRNAs reveal new layers of gene-regulatory complexity, with advances in technologies such as next-generation sequencing and CRISPR, their functions will be gradually deciphered. Of note, recent findings reveal that epitranscriptomic modifications of lncRNAs, such as N6-methyladenosine (m6A), pseudouridine (Ψ), and 5-methylcytosine (m5C), can regulate the diverse functions of lncRNAs [164,165,166,167]. Although epitranscriptomics adds yet another layer of complexity, it may also provide new opportunities for discovering enhanced biomarkers or therapeutic approaches. However, the field of RNA modifications is still in its infancy and much groundwork remains to be done.

In this context, many recent studies have demonstrated the power of diverse CRISPR technologies to decipher the roles of epigenetic modulators, modifiers and mediators in CRC. The CRISPR/Cas9 system has been used for multiplexed knockout screens of epigenetic regulators [47,168]. Another derivate, the CRISPR/deadCas9 system, in which the nuclease activity of Cas9 has been deactivated, can still specifically target any dsDNA sequence through the design of a specific guide RNA. The dead Cas9 system has been adapted to target transcriptional repressors (CRISPRi) and activators (CRISPRa) [169]. Examples are CRISPR/dCas9-VPR for the transcriptional activation of fucosyl transferase (*FUT*) genes [170], DICaS (genome-wide dual protein-coding and non-coding integrated CRISPRa screening) to identify coding and lncRNA genes involved in drug resistance mechanisms [171] and CRISPR/dCas9-KRAB for *HS2* enhancer repression [172,173]. Furthermore, the dead Cas9 system can be used for efficient, directed manipulation of the epigenome via fusion with epi-effector enzymes such as the catalytic domains of TET1 for selective DNA demethylation [174], or DNA methyltransferase DNMT3A for targeted CpG methylation of gene promoters [175]. Dead Cas9 has also been used for targeted localization of lncRNAs to specific genomic loci [176].

Moreover, the CRISPR/Cas9 technology could significantly reduce side effects of epigenetic drugs by targeting them specifically to modulate genes of interest.

Lastly, it requires integrative approaches such as system biology to fully understand gene regulation in CRC by means of epigenetics, alternative splicing, and ncRNAs. The complexity of gene regulation realized by these mechanisms is difficult to comprehend and demands the aid of computer models [177,178]. Spatial models of the intestinal crypts and colorectal cancer development have been reviewed by De Matteis et al. [179]. Recently, a computational model was developed, linking cell surface receptor (EGFR) activation, the MAPK signalling pathway and tumour growth to determine whether ERK inhibitor drugs may be of benefit for CRC patients with the frequently occurring BRAF^V600E^ mutation [180]. Vafaee et al. have integrated data-driven and knowledge-based approaches for biomarker identification in CRC and thereby identified a plasma miRNA signature that is predictive of patients’ survival outcome [181]. To date, however, very few studies have integrated epigenetics and alternative splicing into their models of cancer gene regulation.

## Figures and Tables

**Table 1:** Interplay of ncRNAs with DNA methylation in the pathogenesis of CRC.

Non-Coding RNAs	Epigenetic Partner/Other Epigenetic Mediator	Target Gene	Tumorigenic Effects	Reference
**LncRNA & DNA methylation**				
DACOR1	interaction with DNMT1 to reprogram genome-wide DNA methylation	DNA methylation at thousands of CpG sites	increased clonogenicity	[49,50]
HIF1A-AS2	regulates miR-129-5p and DNMT3A expression		progression and EMT formation of CRC	[56]
H19	hypomethylation of the sixth CTCF-binding site in the differentially methylated region of *IGF2/H19*	loss of imprinting of *IGF2T* → two forms of aberrant IGF2 expression	promotes microsatellite instability and oncogenesis	[62,63,64]
**MicroRNA & DNA methylation**				
miR-133b	promoter hypermethylation	HOXA9/ZEB1 pathway	inhibits migration and apoptosis; suppresses metastasis	[60,65]
miR-149	epigenetically silenced by DNA methylation	Specificity Protein 1 (*SP1*)	independent prognostic factor for overall survival	[66]
miR-132	downregulation by DNA hypermethylation	paxillin	associated with cell invasion	[59]
miR-345	CpG island promoter hypermethylation	BCL2-associated athanogene 3 (*BAG3*)	suppresses colon cancer cell proliferation and invasiveness	[67]
miR-181a/135a/302c	DNA methylation-mediated repression	via repressing PLAG1/IGF2 signalling	promotes the microsatellite-unstable CRC development and 5-FU resistance	[58]
miR-203	directly targets DNMT3B	causes *ABCG2* promoter methylation	predisposing CRC development by lowering expression of ABCG2.	[61]

**Table 2:** Interplay of lncRNAs with other epigenetic partners in the pathogenesis of CRC.

Non-Coding RNAs	Epigenetic Partner/Other Epigenetic Mediator	Target Gene	Tumorigenic Effects	Reference
**Chromatin remodelling**				
DLEU1	recruits SMARCA1, an essential subunit of the NURF chromatin remodelling complex	activation of *KPNA3*	CRC development and progression	[40]
CCAT1-L	regulates long-range chromatin interactions	activates the transcription of the *MYC* locus	both tumorigenesis and the metastatic process	[79]
HOTAIR	reprograms chromatin organization in cooperation with the PRC2 complex	global epigenetic regulation	contributes to liver metastases in stage IV CRC patients	[79]
**Histone modification**				
MALAT1	EZH2	represses E-cadherin	promotes chemoresistance	[80]
HULC	interacts with EZH2	to repress *NKD2*	oncogenic	[81]
SNHG1	interacts with PRC2 in the nucleus and acts as a miR-154-5p sponge in the cytoplasm	modulates histone methylation of *KLF2* and *CDKN2B*	tumour progression	[74]
CRNDE	binds to EZH2	*DUSP5/CDKN1A*	positively correlates with advanced pathological stages and larger tumour sizes	[75]
SNHG17	binds to the EZH2	*p57*	promotes cell proliferation	[82]
SH3PXD2A-AS1	interacts with EZH2	*p57* and *KLF2*	promotes cells proliferation, migration and invasion	[83]
SNHG6	recruits EZH2 to the p21 promoter	*p21*	positively correlates with advanced tumour stage	[84]
MEG3	interacts with PRC2 and JARID2 to direct them to target promoters	Clusterin signalling pathway	inhibits cells proliferation and migration	[85]
PINT	interacts with PRC2 to silence genes	p53 autoregulatory negative mechanism	inhibits proliferation of tumour cells	[86]
PINT	interacts with PRC2	*EGR1*	inhibits tumour cell invasion	[87]
PCAT6	forms a complex with EZH2	activates anti-apoptotic ARC	inhibits colon cancer cell apoptosis	[88]
**Histone modification/DNA methylation**				
Lnc34a	recruits DNMT3A via PHB2 and HDAC1 to methylate and deacetylate the *MIR34A* promoter simultaneously	epigenetically silence miR-34a	Increase colon cancer stem cells (CSCs) proliferation in late-stage CRC s.	[77]
HOXA11-AS	scaffold for the chromatin modification factors PRC2, LSD1, and DNMT1		lymph node metastasis	[89,90]

**Table 3:** List of noncoding RNAs regulating alternative splicing in CRC.

Non-Coding RNAs	Mechanism of Action in AS	Target Gene	Tumorigenic Effect	Reference
LINC01133	titrates SRSF6 away from its targets		inhibits EMT and metastasis	[141]
GAPLINC	binds to PSF and NONO	*SNAI2*	promotes invasion in CRC	[144]
MALAT1	regulates SR splicing factor distribution in nuclear speckle domains		NA	[140]
MALAT1	binds to SFPQ and releases oncogene PTBP2 from the SFPQ/PTBP2 complex		promotes tumour growth and metastasis in CRC	[143]
UXT-AS1	isoform switching from UXT1 to UXT2	*UXT1*	promotes cell proliferation	[146]
miR-1296	represses SFPQ expression	*SFPQ*	accelerates CRC progression	[132]
miR-92a	causing imbalanced expression of PTBP2 through AS-coupled nonsense mediated decay	*RBM4*	contributes to progression and metabolic signature of CRC cells	[133]

## Supplementary Materials

Table S1 and Figure S1 are available online at https://www.mdpi.com/2073-4409/8/8/929/s1. Table S1: LncRNAs sponging microRNAs in CRC. Figure S1: Overview of cross-talk between gene-regulatory layers in CRC (A vector graphics version).
