# Supplementary material for: The Intricate Interplay between Epigenetic Events, Alternative Splicing and Noncoding RNA Deregulation in Colorectal Cancer
Source: Cells. 2019 Aug 19;8(8):929. doi: 10.3390/cells8080929 (PMC6721676; doi:10.3390/cells8080929)
Supplement: Supplementary file 1 [file cells-08-00929-s001.pdf]

Supplementary Materials

Table S1 LncRNAs sponging microRNAs in CRC

LncRNA	Sponged miRNA	miRNA target	Tumorigenic effects	PMID
XIST	miR-124	SGK1 upregulated	doxorubicin resistance	30439718
LINC01410	miR-3128		promotes colon cancer cell proliferation and invasion	30546401
HOTAIR	miR-203a-3p	Wnt/β-catenin signalling	promotes proliferation and chemoresistance	29680837
H19	miR-29b-3p	PGRN/Wnt axis	promotes EMT	29754471
H19	miR-194-5p	FoxM1	promotes EMT	30278464
HOXA11-AS	miR-125a-5p	PADI2	liver metastasis	29050308
MIA-RAB4B	miR-24	RAB4B, PIM2 and TAOK1	contributes to familial adenomatous polyposis-like phenotype	28306719
LINC01503	miR-4492	FOXK1 signalling	promotes CRC cell proliferation and invasion	30542444
LINC00174	miR-1910-3p	TAZ signal pathway	facilitate colorectal carcinoma progression	29729381
FBXL19-AS1	miR-203		proliferation and invasion	28479250
NORAD	miR-202-5p		contributes to proliferation, migration, invasion, and metastasis; poor prognosis	30349308<br>29471886
CRNDE	miR-181a-5p	Wnt/β-catenin signalling	promotes proliferation and chemoresistance	28086904
CRNDE	miR-217	TCF7L2 and Wnt/β-catenin signalling	CRC progression	28472810
TP73-AS1	miR-194	TGFα	promotes proliferation, migration and invasion	30010111
LIFR-AS1	miR-29a	TNFAIP3	resistance to photodynamic therapy	29807108
HEIH	miR-939	NF-κB	promotes CRC tumorigenesis	29081216
CHRF	miR-489	TWIST1/EMT signalling pathway	promotes metastasis	28430582
ABHD11-AS1	miR-133a	SOX4	promotes CRC development	30429229
MALAT1	miR-106b-5p	SLAIN2	promotes the invasion and metastasis	30797712
MALAT1	miR-129-5p	HMGB1	induces colon cancer development	29226325
GAPLINC	miR-34a	c-MET signal pathway	promotes cells migration and invasion	29427222
SNHG6	miR-26a/b and miR-214	EZH2	promotes cell growth, migration, and invasion	30626446
SNHG6	miR-181a-5p	E2F5	CRC progression	30666158
SNHG6	miR-760	FOXC1	CRC progression	30254467
SNHG3	miR-182-5p	upregulating c-Myc and its target genes	promotes CRC progression	28731158

SNHG5	miR-132-3p	CREB5	promotes proliferation, migration and metastasis of CRC cells but inhibits apoptosis	30395767
NEAT1	miR-193a-3p	IL17RD	promotes the tumorigenesis	30407674
NEAT1	miR-193a-3p	KRAS	promotes the tumorigenesis	30575330
GAS5	miR-221		proliferation, migration and invasion	29630521
UCA1	miR-28-5p	HOXB3	proliferation and migration	30652355
UCA1	miR-204-5p	CREB1/BCL2/RAB22A regulatory network	enhances cell proliferation and 5-fluorouracil resistance	27046651
UCA1	miR-143	Cyclin-D1, KRAS and p27	enhances cell proliferation and metastasis	29948578

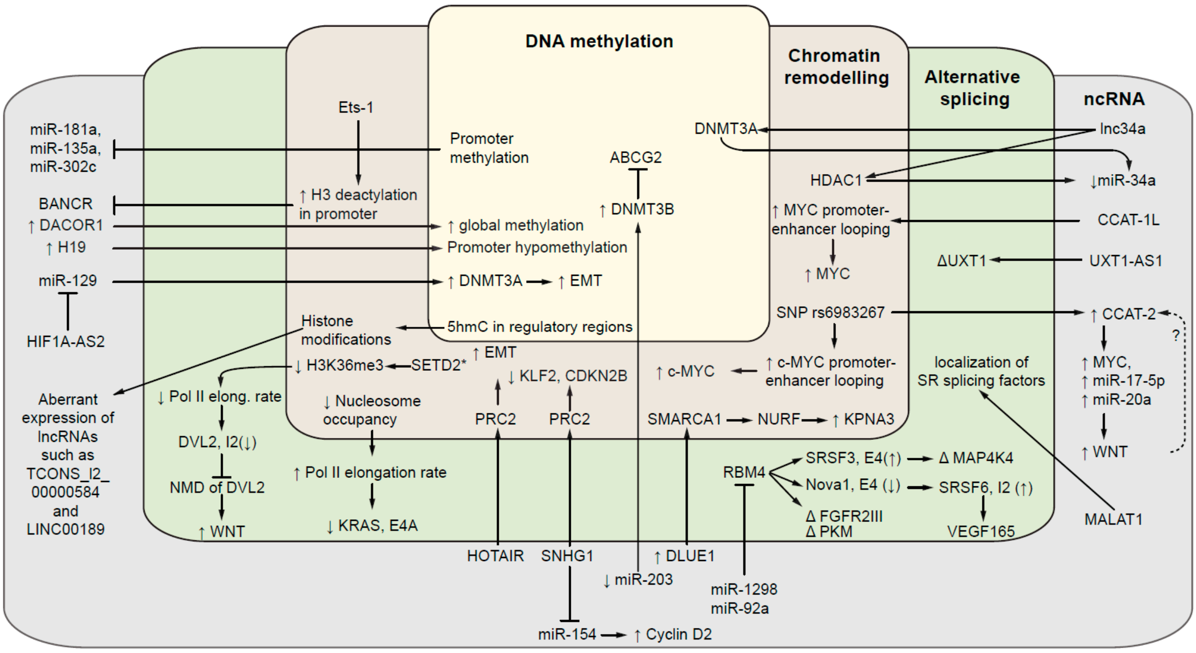

Figure S1. Overview of cross-talk between gene-regulatory layers in CRC (A vector graphics version). This figure depicts selected examples of the deregulated interplay between epigenetic events, alternative splicing (AS) and noncoding RNA in colorectal cancer. See the text of the manuscript for further details. ↑ and ↓ arrows represent up- or down-regulation or higher or lower activity of a factor, respectively. Intron and exons are abbreviated as E or I, respectively. In the cases of AS, ↑ and ↓ represent increased and decreased usage of an exon or intron, respectively. Δ represents isoform switching of a transcript due to AS. SET2D* represents a mutant of SET2D. The dashed arrow with question mark (?) represents a predicted feedback loop between WNT and CCAT-2.
